# A novel controlled release tetrandrine-loaded PDLLA film: evaluation of drug release and anti-adhesion effects in vitro and in vivo

**DOI:** 10.1007/s13346-019-00654-x

**Published:** 2019-06-25

**Authors:** Hai Yao, Zhidong Cao, Lei Peng, Jian Liu, Xiaoxing Zhang, Zhilong Deng

**Affiliations:** Department of Orthopedics, Affiliated Central Hospital, Chongqing University (The Fourth People’s Hospital of Chongqing City), Chongqing, 400014 China

**Keywords:** Tetrandrine, Controlled drug release, Anti-adhesion film, FBSS, Epidural fibrosis

## Abstract

To investigate the drug release and anti-adhesion effects of a TET (tetrandrine)-loaded PDLLA (poly-dl-lactide) film. Detection of TET release in vitro was carried out by high-performance liquid chromatography (HPLC) every 2 days following immersion of the tetrandrine-loaded PDLLA film in simulated body fluid until the TET content of the eluate could not be detected. For the in vivo test, TET-loaded PDLLA films were implanted into animal laminectomy models and positive and blank control groups were also set up. Postoperative serum tests, and macroscopic and histological analyses at 1, 4, 8, and 12 weeks, were used to assess the effects of the film. Statistical analyses were performed by one-way ANOVA. The drug release of the tetrandrine-loaded PDLLA film in vitro showed two phases with a second release peak. Ultimately, the duration of continuous delivery was up to 66 days and the cumulative delivery rate was up to 93.18%. Scores for the proliferation of epidural scars or adhesion of the dura mater in the test group were much lower than those for the two control groups. Histological analysis revealed the test group had fewer inflammatory cells and fibroblasts, as well as fewer extracellular collagen fibers, and a lower histology score than those of the two control groups at all time points. Tetrandrine-loaded PDLLA film is a novel controlled drug release and anti-adhesion material in vitro and in vivo.

## Introduction

Failed back surgery syndrome (FBSS) is a cluster of symptoms following spine surgery, which is characterized by persistent, chronic, or recurrent pain in the back or limbs after laminectomy [1]. Extension of epidural fibrosis into the neural canal and adhesion to the dura mater are among the main causes of FBSS—accounting for up to 24% of all cases—and can lead to chronic nerve radicular pain and lower extremity weakness post-laminectomy [2]. Furthermore, the existence of epidural fibrosis makes revision operation cases more complex. Because of the presence of fibrotic tissue and adhesion at the original surgery site during revision procedures, there is a higher risk of iatrogenic complications such as dural tears, nerve root injury, instability, and adhesion recurrence [3].

Prevention of epidural fibrosis formation has been one of the main concerns for FBSS [4], and numerous materials and methods have been investigated to this end, including developing drugs to reduce inflammation, modifying surgical techniques, using roentgenotherapy, and implanting a biological or synthetic material barrier between the epidural space and the overlying muscles [5–10].

Currently, the use of biodegradable polymeric materials to impede epidural fibrosis adhesion is attracting significant theoretical and practical interest [11]. Poly(d,l-lactic acid) (PDLLA) films have a porous structure that exhibits better physicochemical properties, is more convenient for adding anti-adhesion drugs, and has more controllable degradation compared with traditional non-porous films. DLLA films have been widely used in tissue engineering owing to their excellent biocompatibility and having no obvious toxicity in vivo [12]. Although biodegradable polymeric materials have been shown to significantly reduce epidural fibrosis, the process of re-absorption resulted in a fibrotic mass and a gap forming between the sheet and the dura [13]. Moreover, DLLA alone was unable to prevent nerve root adhesion.

Tetrandrine (TET), which is isolated from the dried root of *Stephania tetrandra* and has been used as a herbal therapy in Chinese medicine for hundreds of years, has been proposed as a therapeutic for the control of scar tissue because of its capacity to inhibit TGF-β1 transcription and its intracellular signaling of hypertrophic scar fibroblasts [14]. However, owing to the hepatotoxicity of TET [15], strategies to reduce the dose of TET in the topical application are required.

In this study, we examined the effect of a novel tetrandrine-loaded poly(d,l-lactic acid) (TET–PDLLA) film as a barrier for impeding fibroblast migration and reducing epidural fibrosis adhesion in a laminectomy rabbit model and investigated the drug release kinetics in vitro and bio-safety in vivo. We hypothesized that the decomposition of the PDLLA film would cause TET to be continuously released and impede scar ingrowths without side effects. The barrier film containing a low concentration of TET would then be safe for use in spinal surgery and may be applicable to future human trials for clinical use.

## Materials and methods

### Drug film preparation and test groups

First, two solutions were prepared. Solution A: 5 g of PDLLA powder was dissolved in 25 mL of dichloromethane. Solution B: 50 mg of TET was dissolved in 0.25 mL of trichloromethane. Second, 25 mL of solution A and 0.25 mL of solution B were mixed to prepare the TET-loaded PDLLA film, with a TET concentration of 0.01%. Third, the concentration of TET was confirmed by high-performance liquid chromatography and found to be 0.0107%. Finally, the solution was transferred into a culture dish to evaporate to give a film at room temperature. The film was cut into squares (length = 4 cm, weight = 503.12 mg (range 482.51–512.41 mg)) and loaded with TET at 10 mg/g to form TET–PDLLA film. The films were sterilized with ethylene oxide before use. The TET–PDLLA film was designated the treatment (T) group; PDLLA film was designated the positive (P) control group, and in the animal experiments, there was also a blank control (BC) group.

### TET release in vitro

Samples of 500 mg of TET–loaded PDLLA film were added to a 20-mL centrifuge tube. After adding 10 mL of simulated body fluid, the centrifuge tube was shaken in a constant temperature shaker at 30 rpm and 37° [16]. Every 2 days, the eluate was collected, an additional 10 mL of SBF was added, and the tube was continually shaken until the level of TET in the eluate could not be detected (lower limit of detection of the instrument 0.2 mg/L). The high-performance liquid chromatography (HPLC) (L2000; Hitachi, Tokyo, Japan) test was repeated five times for every time point, and the average value was used. Finally, a release graph was drawn and the percent TET delivery was calculated.

The chromatographic conditions were as follows: analytical column (4.6 mm × 150 mm, 5 μm), detection wavelength 282 nm, flow velocity 1.0 mL/min, and mobile phase 0.05 mol/L potassium dihydrogen phosphate solution (pH = 3.2)/methanol/acetonitrile (80:30:0.3, *v*/*v*/*v*). A standard curve was created using a TET standard solution, and the regression analysis was carried out by plotting the concentration (*C*) vs peak area ratio (*A*) (*C* = 4.75A + 0.7145 (*r* = 0.9997)).

### Animals

All of the procedures were performed with the permission of the Ethics Committee of Chongqing Medical University, and all of the rabbits were obtained from the Animal Experimental Research Center of Chongqing Medical University. A total of seventy-two New Zealand white rabbits, weighing 4500 ± 270 g, and randomly divided into three groups, were used.

### Surgical procedure

Shortly after anesthesia by intraperitoneal injection of chloral hydrate (2 mL/kg body weight), the hairs around the location of L4 and L5 were shaved, and antisepsis of the exposed skin was performed twice with iodophor. A longitudinal middle incision in the lower back (between L4 and L5 spinous processes) of 3 cm in length was made. After splitting the back muscles bluntly, the two laminae (L4 and L5) were exposed and a laminar defect (3.5 cm × 3 cm) was created to expose the dura mater and nerve roots. The left side of the L4/L5 disk was exposed by retracting the dura mater and nerve roots to the right. The annulus fibrosus was resected and a single “bite” of the nucleus pulposus was made. The laminar defect area was then treated in three different ways: a PDLLA film was cut to a suitable size (4 × 4 cm, ~ 1.25 g PDLLA) and placed over the defect (P group); 1.25-g PDLLA film containing 0.01% TET was implanted on the laminectomy area (T group); the laminectomy area was flushed with saline (BC group). The control and implant sites were randomly assigned in each animal to reduce the influence of any level-specific variations. A standard closure was performed for all animals. No other medical treatment that could reduce the potential effects of the agents was used. Postoperatively, the rabbits were housed in individual cages and allowed normal activity.

### Serum test

Blood was collected preoperatively as well as 1, 4, 8, and 12 weeks postoperatively. Levels of aspartate transferase (AST), alanine transferase (ALT), blood urea nitrogen (BUN), and creatinine (Cr) were measured to assess the effects of the TET–PDLLA film on the function of the liver and kidneys.

### Measurement of the TET concentration in the blood

At the same time points as stated for the serum test, blood samples were drawn from rabbits in the T group and BC group to measure the TET concentration in blood by HPLC.

### Macroscopic observation

Six rabbits were selected randomly from each group and euthanized at 1, 4, 8, and 12 weeks after surgery. The operative area was observed under an operating microscope by a single researcher. The extent of adhesion of each rabbit was scored according to a modified version of the Rydell classification [17].

### Histology and image analyses

At the same time points, as for macroscopic observation, histology samples centered on the vertebral bone, vertebral canal, and its contents, as well as surrounding muscles and scars, were taken from the 6 euthanized rabbits of each group. After staining (hematoxylin and eosin (H&E), van Gieson), a single researcher undertook microscopy and determined histology scores using a modified version of the Nussbaum standard [18]. Simultaneously, H&E-stained sections were analyzed quantitatively using a color image-analysis system (Chinese BUAA CM2000) to determine the area of the epidural scars as well as the ratio of adhesion:non-adhesion of the dura mater.

### Ultrastructural observation

Twelve weeks after surgery, epidural scar tissue taken from each group of rabbits underwent fixing, dehydration, embedding, sectioning, and staining. Variations in ultrastructure were observed using a transmission electron microscope (7500; Hitachi).

### Statistical analyses

Data were analyzed using SPSS v11.0 (IBM, Armonk, NY, USA). Results were reported as *x* ± SD. One-way ANOVA was carried out to analyze differences within and between groups, except for histology scores and macroscopy grades, which were based on 4 × 4 factorial analyses. *p* < 0.05 was considered significant.

#### Data availability

The raw/processed data required to reproduce these findings cannot be shared at this time for legal reasons and because the data form part of an ongoing study.

## Results

### Detection of TET release in vitro

**Fig. 1:**
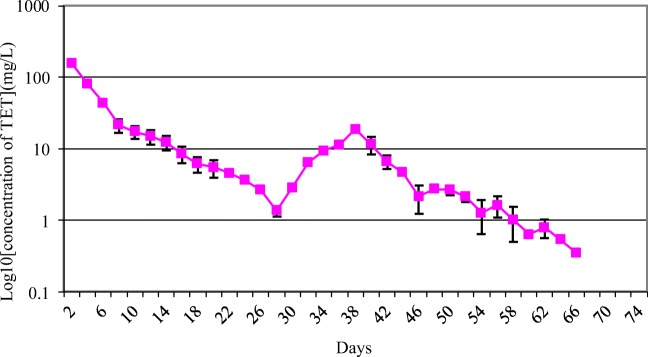
The release curve in vitro of TET-loaded PDLLA film (*n* = 5, mean ± SD). TET, tetrandrine; PDLLA, poly(d,l-lactic acid)

**Fig. 2:**
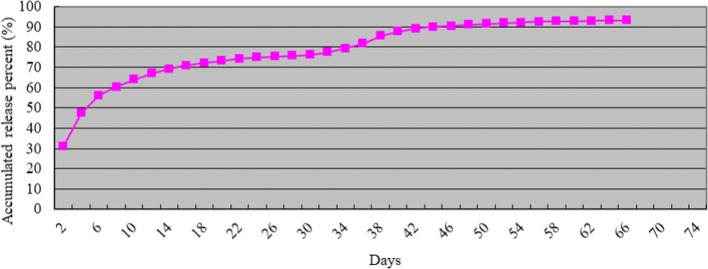
Accumulated release curve in vitro of TET-loaded PDLLA film (*n* = 5, mean ± SD). TET, tetrandrine; PDLLA, poly(d,l-lactic acid)

TET release from the TET–PDLLA film was in three stages: relatively rapid release during the first 48 h, gradual slowing of release until 38 days, and appearance of a second peak (Fig. 1). The duration of continuous delivery was 66 days. Approximately 31.26% of the TET in the TET–PDLLA film was released during the first 48 h, ultimately, the cumulative delivery rate was up to 93.18% (Fig. 2).

### Serum tests

**Fig. 3:**
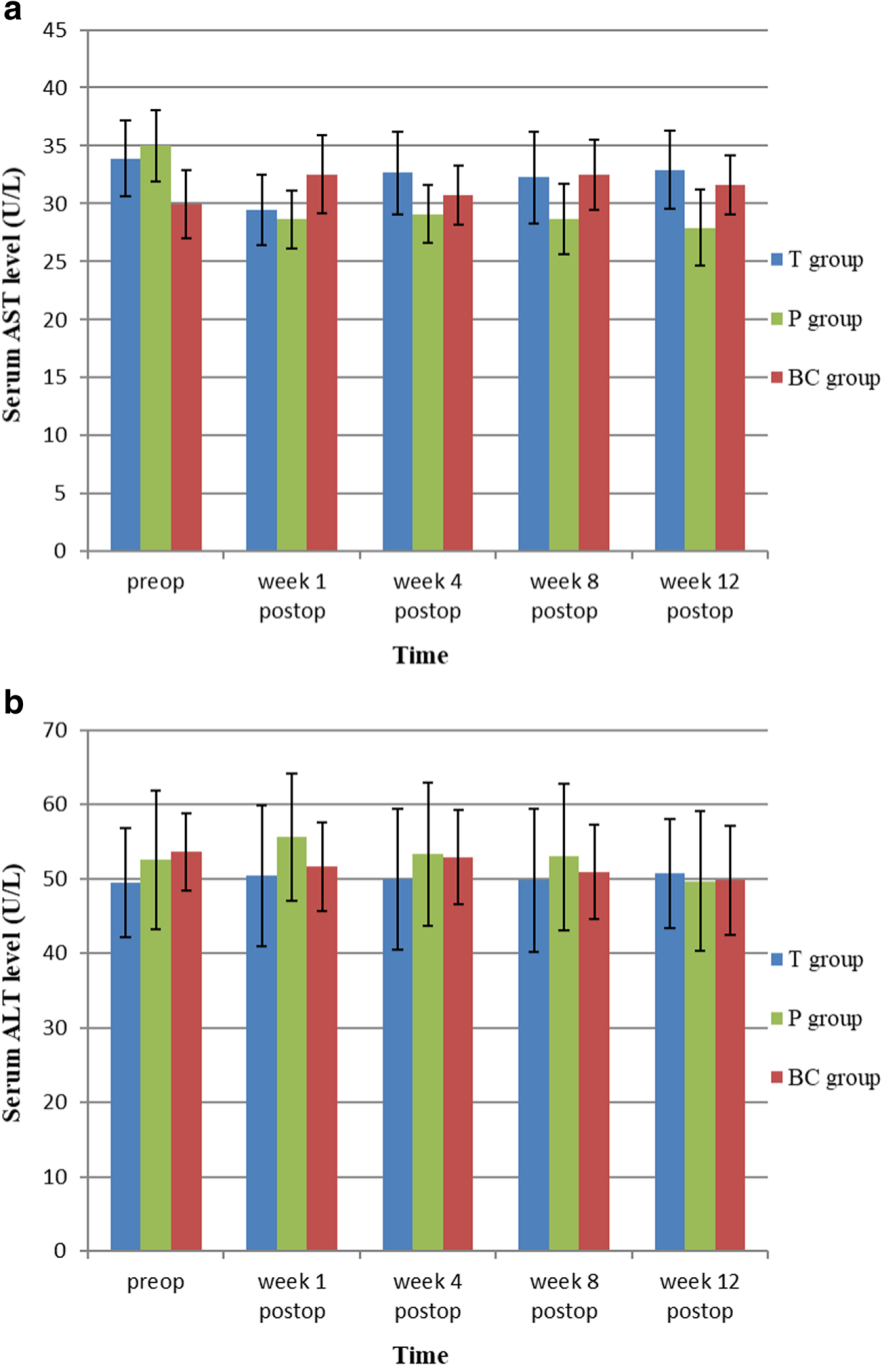
**a** and **b** Effects of TET-loaded PDLLA film on liver function (AST&ALT) (*n* = 24, mean ± SD). TET, tetrandrine; PDLLA, poly(d,l-lactic acid); T group, TET–PDLLA film group; P group, PDLLA membrane group; BC group, blank control group

**Fig. 4:**
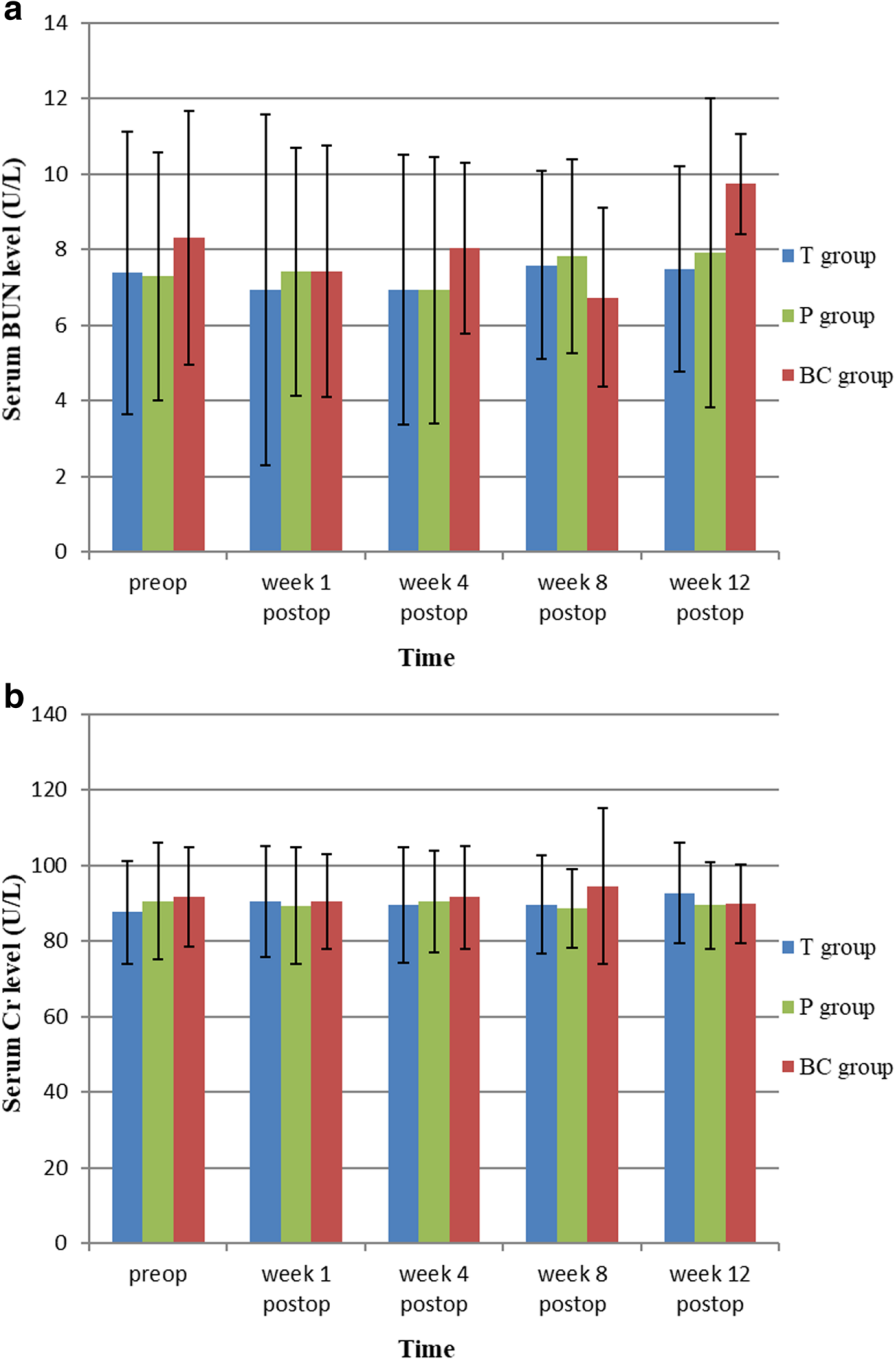
**a** and **b** Effects of TET-loaded PDLLA film on renal function (BUN&Cr) (*n* = 24, mean ± SD). TET, tetrandrine; PDLLA, poly(d,l-lactic acid); T group, TET–PDLLA film group; P group, PDLLA membrane group; BC group, blank control group

The T and P groups both showed no significant difference in liver function (ALT, AST) or renal function (BUN, Cr) compared with the BC group at each time point (Figs. 3a and b and 4a and b).

### Detection of the blood concentration of TET

The postoperative blood concentration of TET in the T group increased gradually until reaching a peak at day 7, followed by a gradual decrease. The postoperative blood concentration of TET at each time point was significantly less than the lethal dose (LD)_50_ of TET (33.3 mg/L [19]) (Table 1).

**Table 1:** Blood concentration of TET (± SD, mg/L)

Groups	Day3 postop	Week 1 postop	Week 4 postop	Week 8 postop	Week 12 postop
T group	< 0.2	8.40 ± 1.58	5.52 ± 1.24	4.53 ± 0.94	2.22 ± 0.22
BC group	< 0.2	< 0.2	< 0.2	< 0.2	< 0.2

< 0.2 means below the detection limit of the instrument (0.2 mg/L). There was a clear difference when compared in pairs of time points at T group (*p* < 0.05)

### Macroscopic observation

**Fig. 5:**
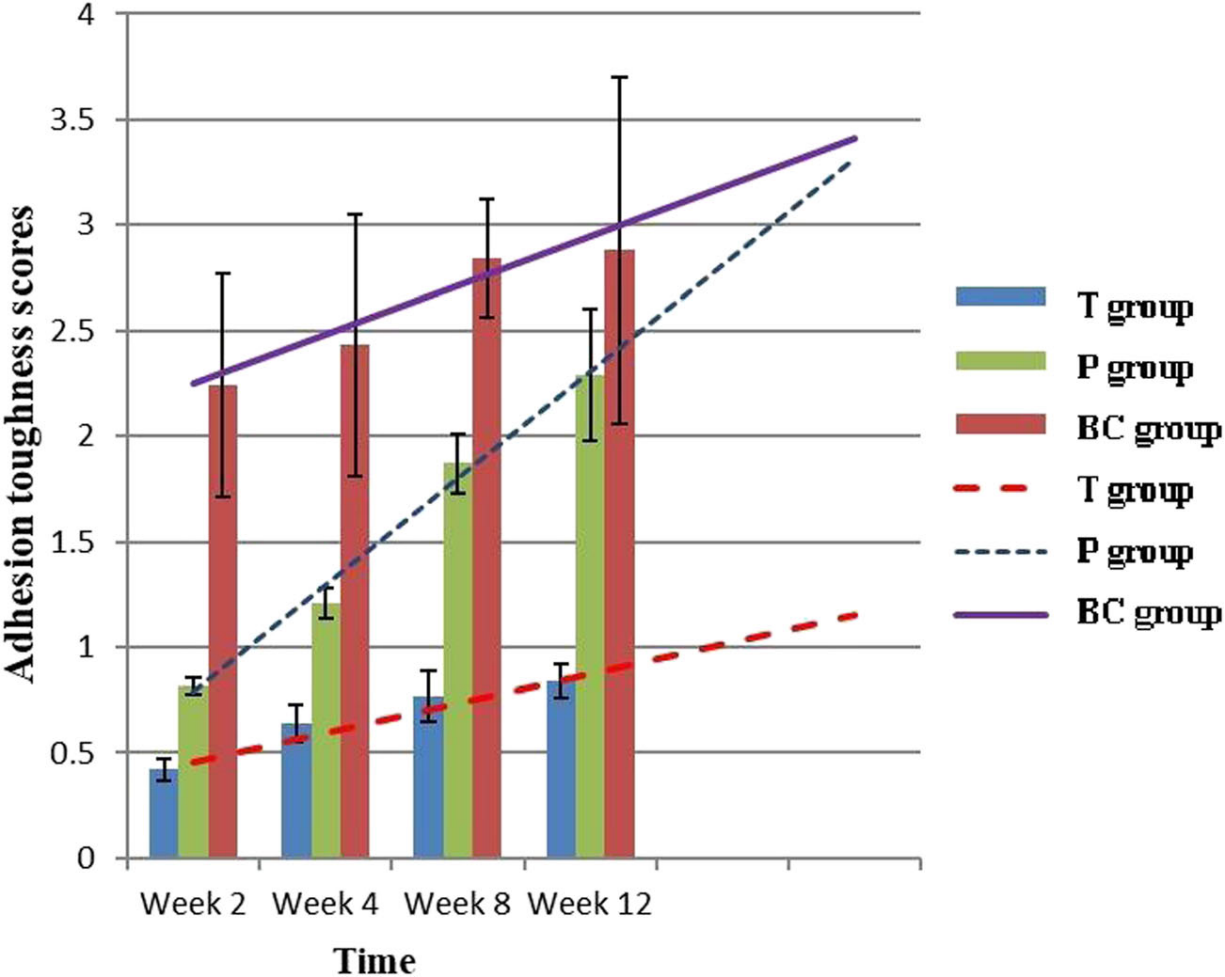
Comparison of epidural adhesion toughness scores among groups (*n* = 6, mean ± SD). TET, tetrandrine; PDLLA, poly(d,l-lactic acid); T group, TET–PDLLA film group; P group, PDLLA membrane group; BC group, blank control group

At each time point, scores for the proliferation of epidural scars or adhesion of the dura mater in the T group were much lower than those of the two control groups (Fig. 5). Four weeks after surgery, posterior adhesion of the dura mater in the P group was similar to that of the T group owing to the barrier formed by the PDLLA film. However, at week 8 or later, after film degradation, posterior and anterior adhesion of the dura mater was worse than that observed in the T group (Figs. 5 and 9).

### Microscopy and image analyses

**Fig. 6:**
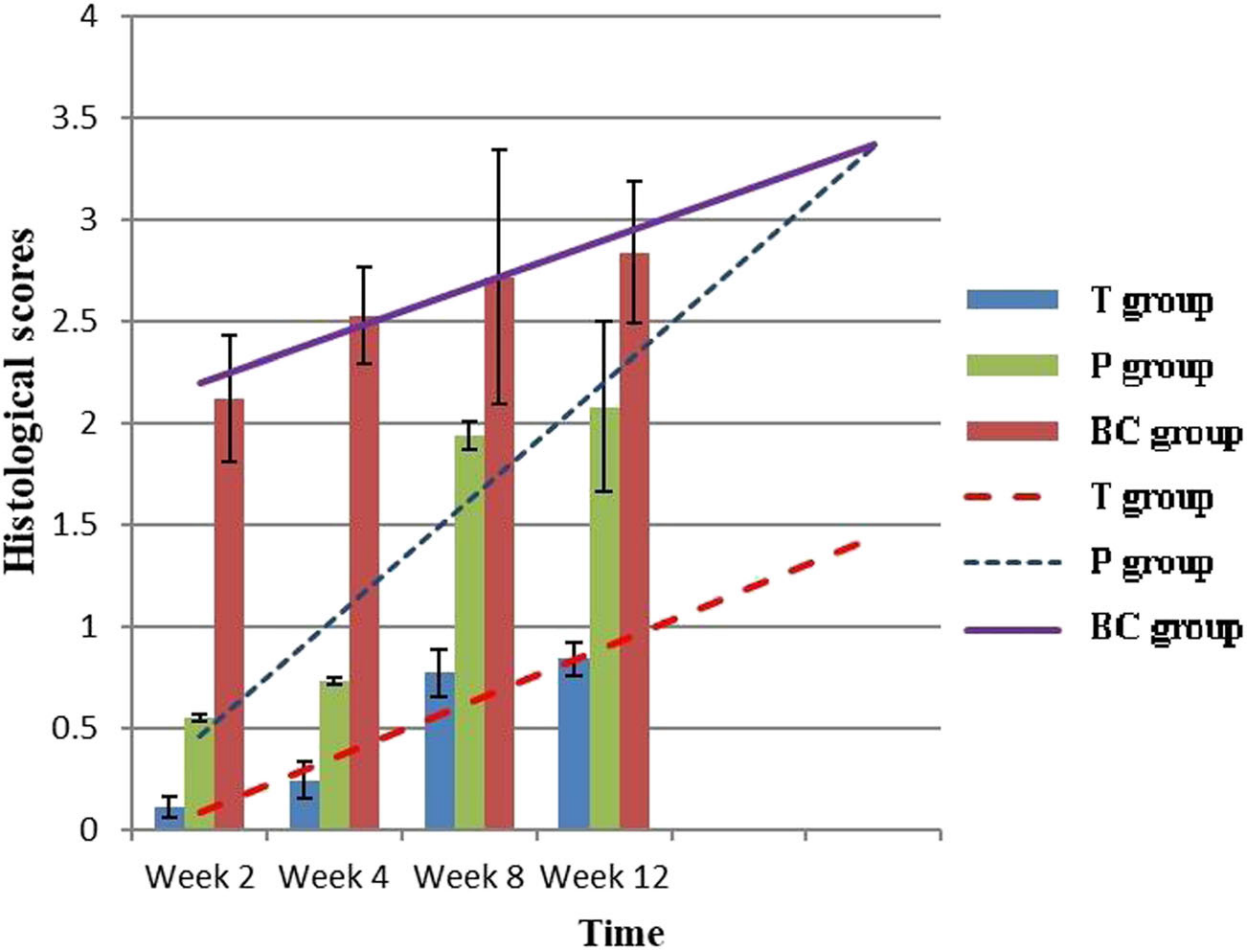
Comparison of epidural histological scores among groups (*n* = 6, mean ± SD). TET, tetrandrine; PDLLA, poly(d,l-lactic acid); T group, TET–PDLLA film group; P group, PDLLA membrane group; BC group, blank control group

**Fig. 7:**
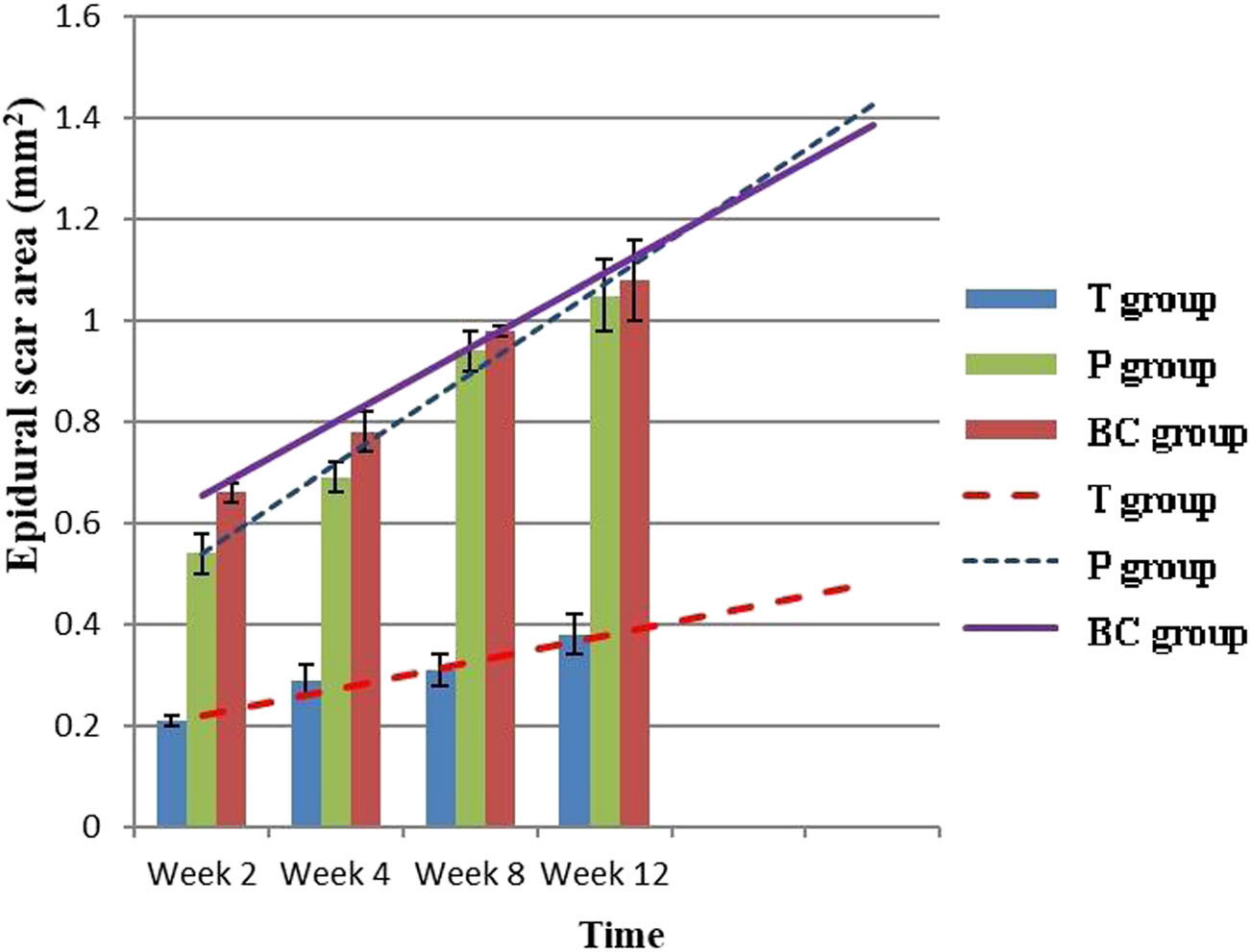
Comparison of epidural scar area among groups (*n* = 6, mean ± SD). TET, tetrandrine; PDLLA, poly(d,l-lactic acid); T group, TET–PDLLA film group; P group, PDLLA membrane group; BC group, blank control group

**Fig. 8:**
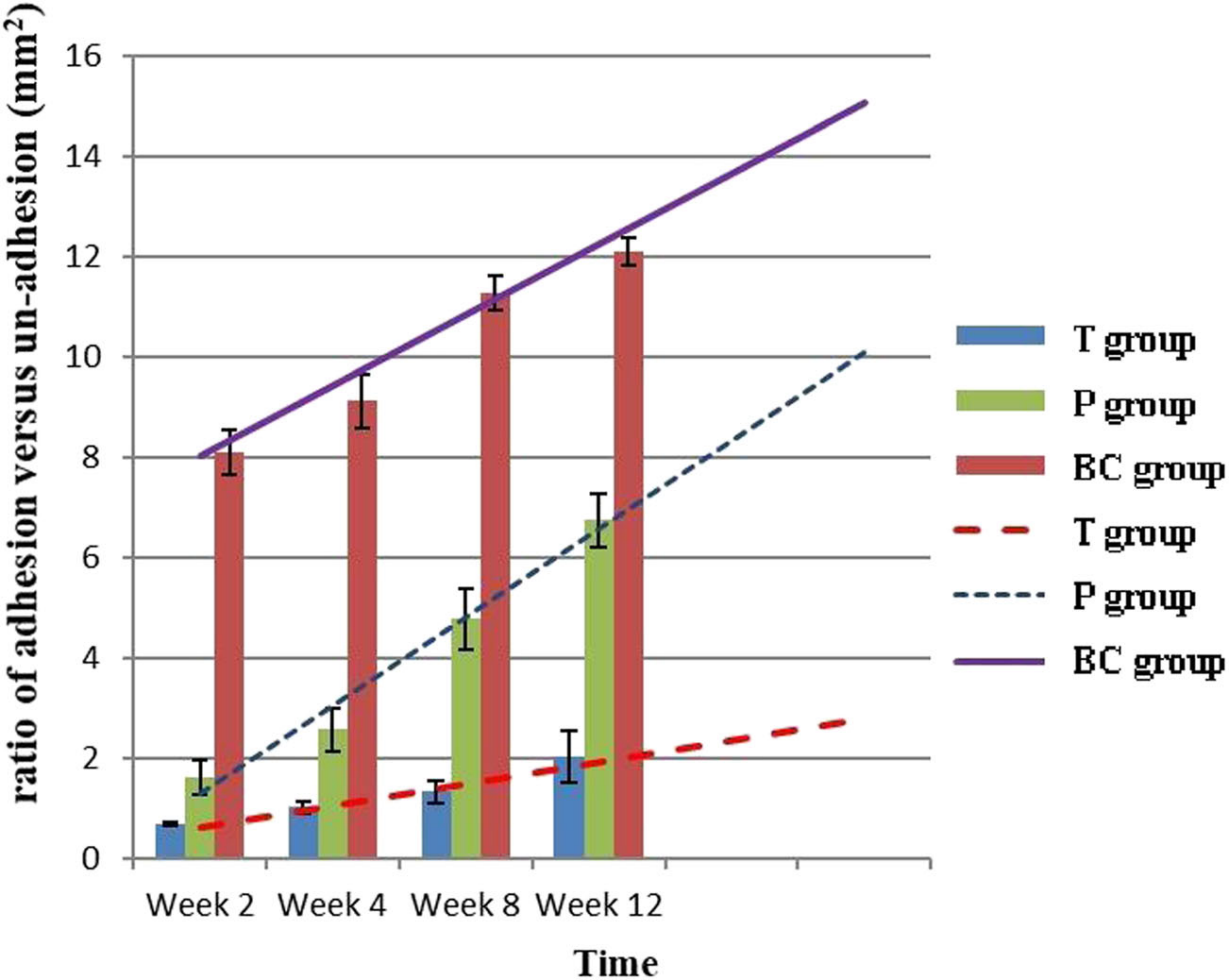
Comparison of adhesion area versus un-adhesion area ratio among groups (*n* = 6, mean ± SD). TET, tetrandrine; PDLLA, poly(d,l-lactic acid); T group, TET–PDLLA film group; P group, PDLLA membrane group; BC group, blank control group

At each time point, histological analysis revealed that the T group had fewer inflammatory cells and fibroblasts, fewer extracellular collagen fibers, and a lower histology score than the P and BC groups (Figs. 6 and 10). Four weeks after surgery, posterior adhesion of the dura mater in the P group was similar to that in the T group owing to the barrier effect of the PDLLA film. However, at week 8 or later, owing to film degradation, the posterior and anterior adhesion of the dura mater was significantly worse than that in the T group. With regard to the area of epidural scarring, as well as the ratio between adhesion and non-adhesion of the dura mater, the T group showed markedly lower values than those of the P and BC groups at each time point (Figs. 7 and 8).

### Ultrastructural observation

**Fig. 9:**
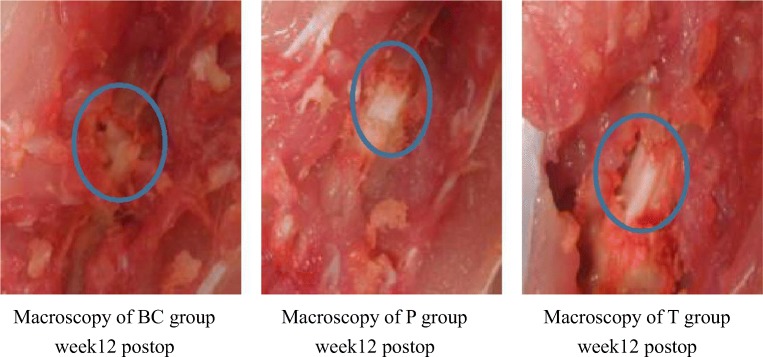
Macroscopy performance week 12 postoperatively. T group, TET–PDLLA film group; P group, PDLLA membrane group; BC group, blank control group

**Fig. 10:**
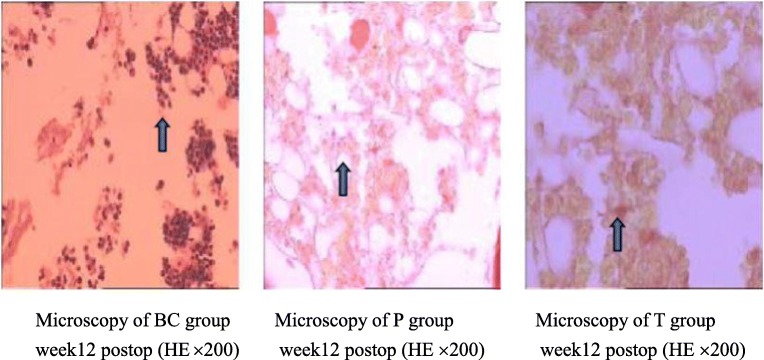
Microscopy performance week 12 postoperatively. T group, TET–PDLLA film group; P group, PDLLA membrane group; BC group, blank control group; HE, hematoxylin-eosin staining

**Fig. 11:**
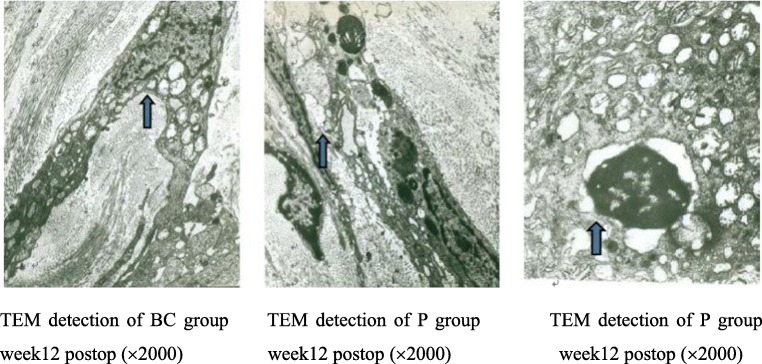
TEM performance week 12 postoperatively. T group, TET–PDLLA film group; P group, PDLLA membrane group; BC group, blank control group; TEM, transmission electron microscope compared in pairs of time points at T group (*p* < 0.05)

Compared with the P group and BC group, the T group showed fewer fibroblasts as well as less cytoplasm, less swelling, or degeneration of mitochondria; fewer rough endoplasmic reticulum in the cytoplasm; deeper staining of the nucleus; and fewer collagen fibers/disorganized collagen fibers in the extracellular matrix. Apoptotic or dying fibroblasts were observed in the T group (Figs. 9, 10, and 11).

## Discussion

For the past three decades, LaRocca’s theory on the “laminectomy membrane” from the posterior surface has guided the research into, and selection and application of, anti-adhesion materials to physically separate the dura mater from posterior scar tissues [20, 21]. However, the posterior barrier cannot prevent anterior adhesion. Hence, materials with the potential to provide protection in all directions were developed, most of which were semifluid. It was reported that these semifluid materials were able to not only protect the dura mater but also “wrap around” nerve roots. Nevertheless, these materials had some drawbacks in clinical use including uneven distribution, short duration of action at the site, and vulnerability to drainage [22, 23]. In addition, some anti-fibrosis drugs and low-dose radiation have been reported to lessen postoperative adhesion by inhibiting the proliferation and function of fibroblasts, which provides a new approach to prevent epidural adhesion [24]. Nevertheless, they have not been applied widely owing to severe systemic toxic effects or an unclear mechanism of action.

TET is a double benzyl quinoline alkaloid extracted from *fourstamen stephania* roots [25]. TET has been used to treat diseases associated with fibrosis within organs, such as pneumosilicosis, liver cirrhosis, and glomerular sclerosis. Previously, we showed that TET can inhibit the proliferation of, and collagen synthesis within, fibroblasts in scar tissue [26, 27]. In addition, TET can prevent epidural adhesion in rabbits to the same extent as that seen for autologous free fat grafts [28].

Owing to its distinct features of biocompatibility, non-toxicity, reliability as a physical barrier, and microporosity, PDLLA was once considered an “ideal” anti-adhesion option [29]. However, PDLLA films cannot prevent anterior adhesion, and its barrier function weakens with degradation. Nevertheless, PDLLA has a prominent advantage in that it can be processed by multiple methods and combined with active drug molecules in various ways. These advantages, in addition to biocompatibility and biodegradability, mean that PDLLA could be used as a controlled release drug-delivery system [30–32].

To reinforce the anti-adhesion effect of PDLLA film, we prepared a novel TET–PDLLA film drug-delivery system, using dissolution and solvent evaporation at room temperature. Our preliminary test found the physical characteristics and mechanical strength of the film to be similar to those of PDLLA film; however, it is possible that processing and sterilization could have introduced side effects and even toxicity. In addition, TET can damage the functions of the liver and kidneys if its in vivo concentration reaches a certain level (LD_50_ = 33.3 mg/L). Therefore, toxicity tests were required before clinical application. In our study, PDLLA film loaded with TET at 10 mg/g did not damage the functions of the liver and kidneys. Moreover, throughout the entire study, the blood concentration of TET was significantly below its LD_50_. These findings may be related to local delivery of TET by the sustained release film, which increased local TET bioavailability but reduced its release into the bloodstream [33]. However, during our preliminary experiments, PDLLA loaded with TET at 20 mg/g was found to cause local soft-tissue necrosis and increase the prevalence of infection without systemic toxicity or damage to the liver or kidneys. These observations showed that the first side effect of TET–PDLLA film was local tissue necrosis and that a film loaded with TET at 10 mg/g would be safe to use in vivo.

A local drug-delivery system can reduce systemic toxicity and increase the local concentration and bioavailability of a drug because it can be designed to release a drug in the desired area for a specific duration [34]. Sustained release anti-adhesion drugs are crucial for epidural adhesion. Ideally, a sustained release anti-adhesion drug should have a high initial rate of delivery (to treat the fibrosis derived from initial surgical trauma and hematomas) and then have an effective concentration over a long time period (to inhibit fiber maturity and contractures) [22]. In this study, TET release from the TET–PDLLA film in vitro was fast during the first 48 h and reached a second peak at day 38; total delivery lasted for approximately 66 days. This three-stage pattern of release was because—as a result of dissolving and solvent evaporation at room temperature—most of the TET was dispersed homogeneously in the PDLLA film; however, some was dispersed on the film surface, which may have led to an initial burst release. Thereafter, TET release was determined by film degradation and drug diffusion. As TET is released, the concentration of TET in the film decreases and TET diffusion also decreases rapidly, so TET delivery could not be maintained for a long time. At this point, increased degradation of the film had a compensatory effect, which resulted in the second peak of TET delivery [18]. It was the second peak of delivery that extended the delivery duration and increased the rate of TET release. Consequently, the duration of TET release reached nearly 66 days and the final amount released was 93.18%, which was in accordance with the criterion of local release of an anti-adhesion drug.

To observe the effects of the TET–PDLLA film on epidural adhesions in vivo, animal laminectomy and diskectomy models were prepared and treated with TET–PDLLA film (PDLLA film and blanks were used as controls). Throughout the 3 months of observation, PDLLA film was found to prevent posterior adhesions only, owing to its film-barrier properties, with no effects on anterior adhesions; moreover, with film degradation, posterior adhesion increased. These results demonstrated again that PDLLA film cannot yet provide prolonged and full protection for the dura mater. In contrast, TET–PDLLA film prevented posterior adhesion, anterior fibrosis, and adhesion, and lowered the ratio of adhesion:non-adhesion of the dura mater and the area of epidural scarring. At each time point, the preventative effects of the TET–PDLLA film were significantly more effective than those of the PDLLA film. In addition, the proliferation and collagen synthesis of fibroblasts were clearly inhibited by the TET–PDLLA film, and the film contributed to the apoptosis and death of fibroblasts as a result of the local role of TET released from the film. Hence, in the early postoperative period, TET–PDLLA film prevented the dura mater from contacting the scar tissue owing to its physical barrier. Subsequently, local TET was released in a sustained manner from the TET–PDLLA film and had a direct impact on fibroblasts (anterior and posterior) by inhibiting proliferation of, and collagen synthesis within, fibroblasts, to reduce scar formation (and shrink the scars already formed).

To address the limitations of our study, further investigations into the optimal anti-adhesion concentration and storage of the film should be carried out, and surface characterization of the film, including wettability and water contact angle measurements, should be performed. In conclusion, TET–PDLLA film is a promising option for preventing epidural adhesion.
